# Disseminating and implementing a lifestyle-based healthy weight program for mothers in a national organization: a study protocol for a cluster randomized trial

**DOI:** 10.1186/s13012-019-0916-0

**Published:** 2019-06-25

**Authors:** Rachel G. Tabak, Cynthia D. Schwarz, Allison Kemner, Kenneth B. Schechtman, Karen Steger-May, Veronda Byrth, Debra Haire-Joshu

**Affiliations:** 1 The Brown School, Washington University in St. Louis, 1 Brookings Dr, St. Louis, MO 63130 USA; 2 Research and Quality, Parents as Teachers, 2228 Ball Drive, St. Louis, MO 63146 USA; 3 Division of Biostatistics, Washington University School of Medicine, Washington University in St. Louis, 660 S. Euclid Ave., CB 8067, St. Louis, MO 63110-1093 USA; 4 The Brown School and The School of Medicine, Washington University in St. Louis, 1 Brookings Dr, St. Louis, MO 63130 USA

## Abstract

**Background:**

Excessive weight gain among young adult women age 18–45 years is an alarming and overlooked trend that must be addressed to reverse the epidemics of obesity and chronic disease. During this vulnerable period, women tend to gain disproportionally large amounts of weight compared to men and to other life periods. Healthy Eating and Active Living Taught at Home (HEALTH) is a lifestyle modification intervention developed in partnership with Parents as Teachers (PAT), a national home visiting, community-based organization with significant reach in this population. HEALTH prevented weight gain, promoted sustained weight loss, and reduced waist circumference. PAT provides parent–child education and services free of charge to nearly 170,000 families through up to 25 free home visits per year until the child enters kindergarten.

**Methods:**

This study extends effectiveness findings with a pragmatic cluster randomized controlled trial to evaluate dissemination and implementation (D&I) of HEALTH across three levels (mother, parent educator, PAT site). The trial will evaluate the effect of HEALTH and the HEALTH training curriculum (implementation strategy) on weight among mothers with overweight and obesity across the USA (*N* = 252 HEALTH; *N* = 252 usual care). Parent educators from 28 existing PAT sites (14 HEALTH, 14 usual care) will receive the HEALTH training curriculum through PAT National Center, using PAT’s existing training infrastructure, as a continuing education opportunity. An extensive evaluation, guided by RE-AIM (Reach, Effectiveness, Adoption, Implementation, Maintenance), will determine implementation outcomes (acceptability, adoption, appropriateness, feasibility, fidelity, and adaptation) at the parent educator level. The Conceptual Framework for Implementation Research will characterize determinants that influence HEALTH D&I at three levels: mother, parent educator, and PAT site to enhance external validity (reach and maintenance).

**Discussion:**

Embedding intervention content within existing delivery channels can help expand the reach of evidence-based interventions. Interventions, which have been adapted, can still be effective even if the effect is reduced and can still achieve population impact by reaching a broader set of the population. The current study will build on this to test not only the effectiveness of HEALTH in real-world PAT implementation nationwide, but also elements critical to D&I, implementation outcomes, and the context for implementation.

**Trial registration:**

https://ClinicalTrials.gov, NCT03758638. Registered 29 November 2018

**Electronic supplementary material:**

The online version of this article (10.1186/s13012-019-0916-0) contains supplementary material, which is available to authorized users.

Contributions to the literature

- Despite efforts at translation, evidence-based interventions often fail to reach young women, who are at high risk for weight gain.
- Healthy Eating and Active Living Taught at Home (HEALTH) is a lifestyle modification intervention developed in partnership with Parents as Teachers (PAT), a national home visiting organization.
- This pragmatic Hybrid Type 2 cluster randomized controlled trial will evaluate the impact of HEALTH on maternal weight and dissemination and implementation of HEALTH across three levels (mother, parent educator, PAT site).

## Introduction

Among women, early adulthood (18–45 years of age) is a particularly vulnerable time period during which they tend to gain disproportionately large amounts of weight, making prevention of weight gain an important target for obesity prevention efforts [1–3]. Maternal weight change in young adulthood, often initiated during pregnancy and maintained after the postpartum period, can impact obesity risk for the rest of a woman’s life [4–6]. Data from the most recent National Health and Nutrition Examination Survey (NHANES) survey show that among adult women, the prevalence of obesity in 2011–2014 ranged from 12% for non-Hispanic Asian women to 57% among non-Hispanic Black women [7], indicating the burden of obesity is borne disproportionately by some groups. NHANES data also demonstrated that obesity increased among women from 2005–2006 to 2013–2014, but not among men [8]. Secular trends toward dramatic gains, particularly in young adult women, have been observed in the few representative longitudinal studies that have been conducted [1, 3, 9, 10]. These trends are also observed in the target population for the current study, with usual care mothers in the trial upon which the current study is based, gaining, on average, 3.2 kg over just 2 years [11]. Reaching young mothers with evidence-based interventions that can reverse the trend of excessive weight gain is critical to reducing the burdens of obesity and chronic disease.

Healthy Eating and Active Living Taught at Home (HEALTH) is an evidence-based intervention, which embeds healthy eating and active living content from the Diabetes Prevention Program (DPP) into the Parents as Teachers (PAT) model [11]. PAT is a national home visiting, community-based organization with significant reach in this population. Dissemination and implementation (D&I) of HEALTH holds promise for impacting the secular trends described above through prevention of weight gain [3]. Follow-up data from the DPP demonstrated the lifestyle intervention, targeting improved dietary intake and physical activity, decreased the incidence of diabetes by 27% over 15 years in overweight and obese adults with impaired glucose tolerance, compared to the placebo group [12, 13]. Improvement in blood pressure can be achieved with as little as 3% weight loss [14–17]. In DPP translation studies, Aziz et al. found the percentage of participants achieving a 5% weight loss ranged from 20 to 64% across interventions [18], and while high-intensity programs had better outcomes in terms of weight loss, low-intensity programs still led to low-to-moderate weight loss and had higher ratings for access and acceptability.

Despite efforts to translate the DPP to broader populations [19–23], evidence-based interventions often fail to reach young women, who are at high risk for weight gain [24]. Women with young children face time and childcare barriers, which impact participation, engagement, and retention; this is of concern as success in programs such as the DPP is associated with attendance [25]. Embedding HEALTH within PAT, which already provides home visiting services, overcomes barriers to participation as mothers are already motivated to participate in PAT to benefit their child, and the program is delivered in the home. This enables participation in HEALTH.

D&I research seeks to bridge the gap of missing critical information about how, when, by whom, and under what circumstances evidence spreads throughout organizations and impacts the target population, identifying key influences on the adoption, implementation, and sustainability of evidence-based interventions [26]. Despite evidence to support the effect of lifestyle interventions for weight management, we were unable to find any D&I studies addressing “if or how” these interventions were integrated within real-world practice settings to have sustainable, broad reach among young women [27]. There was also minimal information on implementation strategies, defined by methods or techniques that might enhance adoption, implementation, and sustainability for lifestyle interventions for young women [26, 28]. The current study goes beyond an effectiveness study, to explicitly evaluate D&I of HEALTH and assess the HEALTH training curriculum (implementation strategy) to understand how to enhance the reach of an evidence-based intervention that prevented excessive weight gain to impact women nationwide. The aims of the study are to (1) determine the effectiveness of HEALTH on weight and behaviors among 252 overweight and obese mothers when disseminated and implemented across 14 real-world PAT sites; (2) evaluate the HEALTH training curriculum’s impact on implementation outcomes (e.g., acceptability, fidelity), at the parent educator level, when parent educators (~ 8 parent educators per site) are trained by PAT National Center; and (3) characterize determinants that influence HEALTH D&I at three levels: mother, parent educator, and PAT site to enhance external validity (reach and maintenance/sustainability).

## Methods

### Overview of the study design

This 5-year study, which began in August 2018, employs a Hybrid Type 2 [29] pragmatic cluster randomized controlled trial to evaluate D&I of HEALTH across three levels (mother, parent educator, PAT site). Since parent educators from each PAT site will be trained together (risk for contamination) and several research questions relate to the PAT site level, a cluster randomized trial, with randomization at the level of the PAT site, is appropriate [30–32]. Further, comparison of HEALTH with usual care PAT (rather than a true control group) is appropriate, as it would not be appropriate to deprive families of the benefit of PAT. This study has been approved by the Human Research Protection Office at Washington University in St. Louis (#201810157).

### Theoretical framework

**Fig. 1:**
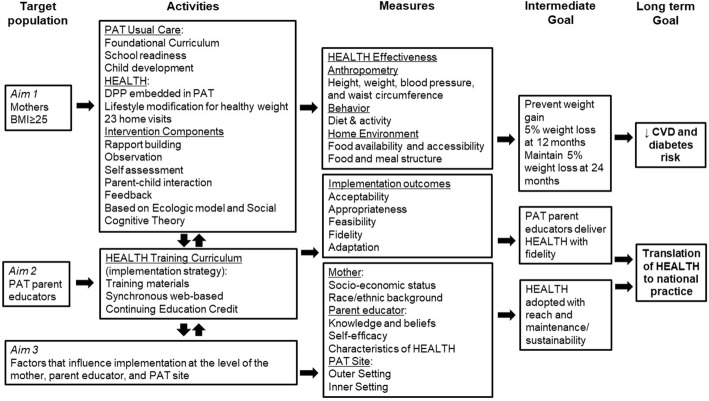
Conceptual model for HEALTH D&I

To inform this study, we combine theoretical perspectives (Fig. 1). We describe an ecologic model that guides the HEALTH intervention which recognizes the protective and interactive influences on young mothers across multiple levels [33, 34]. Social cognitive theory guides the organization of weight loss content, focusing on core behavior change constructs at each level including [1] intrapersonal influences (e.g., constructs of self-assessment, reinforcement, and behavioral capability) [2], interpersonal influences (e.g., observational learning/parental model for child), and [3] how these interactions influence, or are influenced by, the environment of the mother (e.g., home environment) [33–35].

Two frameworks from D&I science also inform our study. The Consolidated Framework for Implementation Research (CFIR) helps identify determinants, or contextual factors, that impact D&I of an intervention [36]. We will employ CFIR to guide our characterization of determinants that influence HEALTH implementation at three levels: mother, parent educator, and PAT site, to enhance research translation [37, 38]. This model offers a menu of constructs that can guide preparation for and improvement of D&I through a systematic assessment of the implementation context, such as potential barriers and facilitators. CFIR constructs are drawn from a wide-ranging evidence base [39, 40] and have been associated with effective implementation [37, 38].

To guide our evaluation of implementation outcomes and external validity, we will use a second framework from D&I science, RE-AIM (Reach, Efficacy, Adoption, Implementation, Maintenance), the leading framework for evaluating the applicability of interventions in practice settings [41–45]. Reach refers to the number, proportion, and representativeness of those who participate in the intervention relative to all persons in the target population [46, 47]. Efficacy (and effectiveness) identifies effects of an intervention [42, 43, 46, 48, 49]. Adoption refers to the absolute number, proportion, and representativeness of settings (PAT sites) and agents (parent educators) that agree to implement the intervention and is influenced by core elements of appropriateness and feasibility [42, 47, 48, 50]. Implementation involves implementation outcomes related to delivering the intervention as intended (i.e., acceptability, fidelity, adaptation). The Framework to Documenting Changes to Programs developed by Wiltsey Stirman et al. [51] will be used to organize adaptations made to the intervention when implemented as part of usual practice. Maintenance refers to sustained behavior change over time among participants, and integration or institutionalization of the intervention as a routine organizational practice [44, 52, 53].

### Setting and participants

Sites will be selected from the pool of 3246 sites to be among the 28 included in the trial then randomized to usual care (*N* = 14) or HEALTH (*N* = 14). To be eligible, sites must report seeing 50 families per year, to assure the required number of mothers per site will be able to be recruited during the study period. Further, to avoid compromising a site’s ability to complete its primary function of delivering PAT by imposing the burden of a study, only sites in compliance with the PAT model are eligible. From sites agreeing to participate, all educators will be offered the HEALTH training curriculum, with those randomized to HEALTH, receiving it at the beginning of the trial, and those randomized to usual care, receiving it at the end of the study. The number of educators per site ranges from 3 to 50; based on our previous work, we anticipate an average of eight parent educators to be trained per site. Given the sample size requirement of 12 sites in each arm providing complete data, we will recruit 14 sites per arm to allow for site-level attrition.

### Mother recruitment, eligibility, and screening

We will recruit an average of 18 mothers per site. Our plan to recruit mothers mirrors routine PAT “rolling” recruitment throughout the year. Parent educators will provide study fliers to families (parent educators commonly bring materials to families). Interested families will either call the number on the flyer or indicate their interest to the parent educator; the parent educator will send the names of mothers who express interest in additional information to the study team staff who will coordinate an outreach/screening call. These staff will screen potential mothers by phone to ascertain self-reported height and weight, along with other eligibility criteria (Table 1). If eligible, the staff member will schedule baseline data collection. At the home visit, the staff member will consent the mother to the study and assess height and weight to assure eligibility. This process will continue until 18 mothers have been enrolled from each site. Those not providing consent will continue with PAT visits, but will not participate in data collection or be part of the study.

**Table 1:** Inclusion and exclusion criteria for mothers

Criteria	Inclusion	Exclusion	Assessment
Age	18–45 years		Self-report
Obesity status	BMI = 25–45 kg/m^2^		Measured on scale/stadiometers
Pregnancy status	Not pregnant	Less than 6 months postpartum; Planning to become pregnant in the next 24 months	Self-report
PAT participation	Participating in or willing to participate in PAT	Planning to move out of the service area of the PAT site or to stop participating in PAT in the next 24 months	Self-report
Research participation	Willing to provide informed consent; agree to all study procedures and assessments; speak English or Spanish	Unable to provide informed consent, unable to engage in a walking program	Self-report

As a pragmatic trial, it is important that mothers in the study closely match the usual case load for PAT; inclusion criteria have been selected accordingly and are described in Table 1 [54]. Any changes to these criteria will be reflected in a modification to the IRB and to the protocol.

### Randomization

PAT sites will be randomly assigned to HEALTH or usual care via a dynamic randomization scheme [55], in which SES (i.e., percent of low-income families) and number of families served will be considered so that we achieve similar distributions in both arms of the study. The first 8–12 sites will be recruited and randomized at the same time to HEALTH or usual care arms in a 1:1 ratio within site pairs where two sites are matched on size and SES. Subsequent sites will use a dynamic randomization scheme to maintain the balance of SES and size between the two treatment groups. This two-tiered recruitment is for both pragmatic reasons (to avoid training all sites at once) and to ensure a more balanced sample between groups. Due to the nature of the intervention, this study is not blinded.

### Intervention conditions

PAT National Center, located in St. Louis, Missouri, is responsible for training and certifying all parent educators to deliver the PAT model (e.g., use of Foundational curriculum [56], adherence to model fidelity requirements [57], and other specialty trainings). Trainings take place in person, and more recently, specialty trainings have been offered via extensive web-based facilities to assure national reach. Parent educators maintain their certification through annual continuing education offered by PAT National Center. Through our partnership with PAT National Center, parent educators will receive continuing education credits for completing the HEALTH training curriculum.

Mothers at usual care PAT sites will receive the Foundational (usual care) curriculum that uses a strength-based, solution-focused model [56]. It includes 60-min home visit plans [56] that focus on (1) rapport building and relationship building (e.g., reflective questions to get to know the family such as “What did you notice about your child’s development since the last time we met?”); (2) development-centered parenting, which is the relationship between the parent’s understanding of their child’s development looking at specific developmental topics (attachment, safety, sleep, discipline, transitions/routines, health, nutrition) and parenting behaviors (nurturing, designing/guiding, responding, communicating, and supporting learning); (3) parent–child interaction, which is focused on supporting parents to engage in different types of positive interactions with their child and the parent will observe and learn about their child’s personality and temperament (e.g., measuring dried beans, reading together); (4) family well-being, which is focused on understanding that the family system that can impact the parent and child and connecting the family to resources, where needed (e.g., address needs in the family’s physical and emotional environment such as “Who do you think supports your child to be happy and successful?”); and (5) closing/reinforcement and follow-up plans (e.g., feedback on goals, praise for success, topics the parent wants to address on their next home visit). The frequency and number of visits are based on the family’s needs and preferences.

Mothers at HEALTH (intervention) sites receive the Foundational curriculum + evidence-based lifestyle change strategies to prevent weight gain and promote weight loss embedded within and delivered as part of home visits. The HEALTH intervention is guided by PAT’s strength-based, solution-focused model plus a socio-ecologic approach recognizing the protective and interactive influences on women across multiple levels (see the “Theoretical framework” section) [33, 34]. Additional file 1: Table S1 outlines the topics, intents, and prompts/questions for each visit in two phases of the intervention (i.e., core and maintenance visits described below). Goal setting related to healthy weight, and the importance of parental modeling of healthy eating and physical activity are incorporated within the discussion and visit. The lifestyle modification content is simplified to focus on the behaviors most likely to impact weight through calorie intake and physical activity. Dietary behaviors include limiting intake of sugar-sweetened beverages, substituting fruits and vegetables for high caloric snacks, limiting portion sizes; activity targets are increasing physical activity by walking more and decreasing sedentary activity, such as TV viewing. To make the HEALTH content feasible to deliver and sustainable within the PAT model, it was designed to fit within the existing family well-being component of the visit.

### Visit structure/dose

HEALTH will be delivered over 24 months via a [1] core and [2] maintenance phase. Modeled after the DPP [12, 13, 58] and various effective DPP adaptations [21, 23], the visits begin with greater frequency, and taper. The core phase is the most intense and structured phase, delivered via ~ 8 home visits. During this phase, mothers are taught basic information about healthy eating and physical activity and are given the opportunity to practice related behavioral skills during home visits. The maintenance phase reinforces lessons learned in the core phase while assisting mothers in focusing on issues of relevance and problem-solving via 15 home visits.

### HEALTH training curriculum (implementation strategy)

As parent educators are from real-world PAT sites, all (HEALTH and usual care) were trained in Foundational Curriculum, Model Implementation. To implement the lifestyle modification content, we used Powell et al.’s compilation of implementation strategies to select discrete strategies to build a tailored multicomponent strategy for implementation of HEALTH [59] to *develop* and *distribute HEALTH educational materials*, *make parent educator training dynamic*, and *provide ongoing consultation*. The dynamic training will be delivered through two half-day (4-h) interactive trainings. Consistent with PAT training protocols we have successfully implemented [60–62], HEALTH educators are offered a pretest and posttest reflecting training objectives. Finally, ongoing consultation will include “booster” sessions (ongoing consultation), provided via phone or video conference, consistent with PAT “communities of practice.” Parent educators will receive training and supervision by the project manager (dietitian and PAT parent educator) and by a parent educator with experience in similar trials. These sessions will be scheduled approximately monthly for 6 months after initial training and taper over the following 6 months. Parent educators will receive continuing education credits (required by PAT for all parent educators) for participating in the HEALTH training curriculum. The development of the training materials, to be consistent with usual PAT practice, is described below; these will be distributed to sites by mail and/or electronically.

### Development of embedded intervention and implementation strategy

Both the lifestyle intervention (HEALTH) and the implementation strategy to embed the intervention (HEALTH training curriculum) were developed in partnership with PAT to allow for improved fit and to enhance the potential for sustainability. Formative research with PAT staff ensured HEALTH is consistent with PAT’s mission, practice, and funding requirements. To align the HEALTH visits with usual care, PAT parent–child activities were incorporated into HEALTH content, and all materials were designed to be consistent with parent and parent–educator facing materials.

To embed the HEALTH training curriculum within PAT, we developed the implementation strategy to be delivered through PAT’s existing infrastructure [59]. Consistent PAT practice, PAT parent educators will be trained to deliver HEALTH through a specialty training delivered by PAT National Center through a synchronous web-based experience. We worked with PAT National Center to harmonize the HEALTH training curriculum with the organization’s usual practice and training infrastructure.

## Study measures

### Effectiveness outcomes

The impact of HEALTH on maternal weight and weight behaviors over 12 and 24 months, relative to usual care PAT, will be assessed by anthropometry, blood pressure, behavior measures, and home environment measures. These measures are summarized in Table 2.

**Table 2:** Summary of aim 1 mother-level measures

	Collection method		Time point		
	Data collector	Self-Report	Baseline	12 months	24 months
Demographic and socio-economic characteristics		●	●		
Anthropometrics					
Height	●		●		
Weight	●		●	●	●
Blood pressure	●		●	●	●
Waist circumference	●		●	●	●
Safety monitoring (excessive weight loss ≥ 15%)	●				
Behavior					
NHANES Dietary Screener Questionnaire [11]		●	●	●	●
IPAQ [63]		●	●	●	●
Home environment					
Influence of home food environments on eating behaviors [64]		●	●	●	●
Individual parent-level satisfaction					
Measure (RE-AIM measure: acceptability)		●		●	●

Maternal height will be measured at baseline; maternal weight, blood pressure, and waist circumference, as well as the home environment, will be measured at baseline, 12-month, and 24-month follow-up, in the mother’s home or in an alternate, private location (e.g., library, doctor’s office). Each site will be provided with appropriate equipment (e.g., scale, stadiometer, tablet), which data collectors will bring to measurement visits.

Primary outcome measures: A portable stadiometer (Seca 213 stadiometer) and standardized protocol will be used to assess height to the nearest 1/8 in. Weight will be assessed to the nearest 0.1 lb. using an electronic scale (Health-o-meter 349KLX digital scale). Attempts will be made to weigh mothers at about the same time of day for each measurement, after the mother empties her bladder and removes shoes and excess clothing [65].

Secondary outcome measures: Waist circumference will be used to estimate abdominal adiposity which is associated with risk of cardiovascular disease and diabetes [66–68]. Furthermore, national findings underscore the importance of considering waist circumference values among certain racial/ethnic groups [67]. Based on these data, we will include age, sex, and race-/ethnicity-specific waist circumference percentiles in our analyses (i.e., Caucasian and African–American) [67, 69]. To enhance standardization across data collectors and sites, blood pressure will be measured with OMRON Model HEM-907XL automated cuff, which has been used in previous studies [70, 71]. Assessments of behavior will include dietary change as measured by the NHANES Dietary Screener Questionnaire, which asks parents to recall frequency of intake of foods and drinks over the prior month [72]. Physical activity will be assessed with the International Physical Activity Questionnaires (IPAQ) [63] using a seven-item activity module that measures sedentary behavior and general type, frequency, duration, and intensity of physical activity with (low, moderate, or high intensity) category scores. The home environment will be measured by parent report using the Household Food Practices and Family Social Support items developed by Kegler et al. [64]. Mothers will receive $20 for each research visit.

### Implementation outcomes and external validity

Qualitative and quantitative assessments of implementation outcomes and external validity (Table 3) within the RE-AIM framework allow us to better understand the implementation outcomes and context, building a deeper understanding of how to maximize HEALTH D&I and impact [73–76, 79].

**Table 3:** RE-AIM constructs and measures for HEALTH practice outcomes and external validity

RE-AIM outcome	Definition	Level	Sample item/citation
Reach/representativeness (aim 3)	Absolute number, proportion, and representativeness of individuals who participate in HEALTH	• PAT site	Calculated from administrative data.
Effectiveness (aim 1)	Impact of HEALTH on weight and important lifestyle behaviors (e.g., diet and activity)	• Individual mother	See Table 1.
Adoption (aim 2)	Intention, initial decision, or action to try or employ HEALTH; “uptake”	• PAT site	Calculated from PAT National Center administrative data. *I would try a new curriculum even if it were very different from what I am used to doing* [73]*.*
Appropriateness^a^	Perceived fit, relevance, and compatibility of HEALTH for PAT and parent educators; and perceived fit of HEALTH to address weight	• Parent educator	*I do not know why promoting healthy weight behaviors is so important for PAT to address* [74, 75]*.*
Feasibility^a^	Extent to which HEALTH can be successfully used or carried out within a given agency or setting	• Parent educator	*HEALTH is easy to implement correctly at this PAT site* [74, 75]*.*
Implementation (aim 2)			
Acceptability^a^	Perception among implementation stakeholders that HEALTH is agreeable, palatable, or satisfactory	• Individual mother • Parent educator • PAT site	*Using HEALTH will enhance my effectiveness on the job* [73, 76]*.*
Fidelity	Degree to which HEALTH was implemented as prescribed in the original protocol or as it was intended by the program developers	• Parent educator	*How much of the lesson plan content was delivered?* Visit checklist and recorded home visits.
Adaptation^a^	Planned or purposeful changes and unintentional deviations to the design or delivery of HEALTH	• Parent educator	*Have you made any changes to address client (family)-level needs, preferences, or constraints?* [53, 77, 78] Recorded home visits.
Maintenance/sustainability (aim 3)	Extent to which HEALTH is maintained or institutionalized within PAT’s ongoing, stable operations	• PAT site • PAT National Center	*The program is well integrated into the operations of the organization* [74, 75]*.*

^a^Includes qualitative measures

The impact of the HEALTH training curriculum will be assessed by qualitative and quantitative measures of appropriateness [74, 75, 79, 80] and feasibility [74, 75, 79, 80] within the Adoption domain; three outcomes in the Implementation domain will also be assessed: acceptability, fidelity, and adaptation. Acceptability will be assessed qualitatively and quantitatively [73–76, 79] at the level of the mother, parent educator, and PAT site. Measures of fidelity will be calculated from two data sources from parent educators at usual care PAT and HEALTH sites: (1) home visit records and (2) recorded home visits. Specifically, parent educators will complete home visit records, documenting adherence, quality of delivery, exposure to the intervention, and participant responsiveness or involvement [81–84]. Parent educators will also audio record visits for which the mother consents to recording; project staff will review a subset of 2 visits for 2 parent educators per site. Qualitative interviews and visit audio recordings will capture and categorize adaptations to HEALTH and the reasons for the adaptation (e.g., cultural factors), using methods developed by Wiltsey Stirman et al. [51, 85, 86] and recommended by others [84, 87–90].

External validity (reach, adoption, and maintenance) will be determined using administrative and survey data. PAT sites report service delivery (e.g., tracking personal visits) and administrative data including characteristics (e.g., high needs characteristics) of the families they serve, to PAT National Center annually through digital reporting systems. This will allow for comparison of HEALTH mothers with typical PAT mothers using participation rates to determine the representativeness of participating mothers and sites. Adoption and maintenance will be assessed by a survey [53, 73, 77, 78].

### Context assessments

CFIR was selected as the framework to guide the assessment of determinants influencing HEALTH implementation due, in part, to the extensive work to establish and organize measures for its constructs (Table 4). Determinants at the level of the mother will be assessed with measures from the HEALTH effectiveness trial to measure socio-economic status and racial/ethnic background and a measure of nutrition and activity cultural norms [91, 95]. To assess parent educator characteristics, the parent educators will report their knowledge and beliefs about HEALTH [80] and their confidence (self-efficacy) [74, 75] in delivering HEALTH. Parent educators will be asked their perceptions of HEALTH, to determine characteristics of the intervention (e.g., complexity, relative advantage) [80, 92]. At the PAT site level, outer setting factors will be assessed using survey, administrative, and service delivery data. Questionnaires will assess site leader and parent educator perception of the PAT site’s inner setting: culture, implementation climate, learning climate, leadership engagement, and available resources [93] as well as readiness to implement HEALTH [94]. The administrative and service delivery data PAT sites annually report (e.g., tracking personal visits, high needs characteristics) to PAT National Center provide structural characteristics about the PAT site (e.g., number of families served per year).

**Table 4:** Consolidated Framework for Implementation Research (CFIR) determinants that influence HEALTH implementation (aims 2 and 3)

Factor	Definition	Sample item/citation
Mother level		
Socio-economic status	Social standing or class of an individual or group	*Which of the following describes your current living situation?* [11]
Racial/ethnic background	Participant’s geographic region of origin, ancestry and cultural tradition, common history, religion	*Which of the following represent your Hispanic origin or ancestry?* [11]
Cultural norms	Unspoken rules or patterns of behavior that are perceived as normal or socially acceptable	*Most people who are important to me think I should make healthy food choices* [91]
Parent educator level		
Parent educator characteristics		
Knowledge and beliefs about HEALTH	Parent educators’ attitudes toward and value placed on HEALTH and familiarity with facts, truths, and principles related to HEALTH	*I can distinguish between different curricula which address healthy weight* [80]*.*
Self-efficacy	Parent educator belief in their own capabilities to achieve implementation goals	*I am confident I can implement HEALTH as prescribed at this PAT site* [74, 75]*.*
Intervention characteristics—HEALTH		
Evidence strength and quality	Parent educators’ perceptions of the quality and validity of evidence supporting the belief that HEALTH will have desired outcomes	*HEALTH should be effective, based on current scientific knowledge* [92]*.*
Relative advantage	Parent educators’ perception of the advantage of implementing HEALTH versus usual care	*In general, HEALTH would be more effective in helping women prevent gaining weight than our current curriculum* [80]*.*
Complexity	Perceived difficulty of implementation, reflected by duration, scope, radicalness, disruptiveness, centrality, intricacy, and number of steps required	*HEALTH would be difficult to teach* [80]*.*
PAT site level		
Outer setting		
Participant needs and resources	Extent to which client needs (and barriers and facilitators to meeting those needs) are accurately known and prioritized by the organization	*HEALTH will improve the overall quality of life for clients who receive it.*
External policy and incentives	External strategies to spread interventions, including policy and regulations (governmental, other central entity), external mandates, recommendations and guidelines	*Do you anticipate any funding changes in the next few years?*
Inner setting—PAT site		
Structural characteristics	Social architecture, age, maturity, and size of a PAT site	Determined from administrative data.
Culture	Norms, values, and basic assumptions of a PAT site	*Management at your PAT site reward innovation and creativity to improve practice client care* [93]*.*
Implementation climate	Absorptive capacity for change, shared receptivity of involved individuals to an intervention, and the extent to which use of the intervention will be rewarded, supported, and expected in the PAT site	*Staff members involved in implementing HEALTH are appreciated for their efforts* [93]*.*
Readiness for implementation	Tangible, immediate indicators of organizational commitment to its decision to implement HEALTH	*People who work here are determined to implement this change* [94]*.*

### Quantitative data collection procedures

Based on the recommendation of our PAT partners, parent educators will not conduct screening or data collection; rather, the research team will provide two options for data collection. One option is to hire and train data collectors located near the participating PAT sites; the second is to send staff to the sites to complete data collection. Data collectors will be hired through organizations that employ individuals with experience conducting home visits and taking measurements. To improve response rates and minimize missed visits, data collectors will coordinate with parent educators to reach and meet families. Study staff will conduct a site visit in addition to video conference training on study procedures, measures, and completing consent. Data collectors will contact mothers by phone prior to measurement visits so visits are not missed. As study team members, data collectors will complete human subject certification.

PAT site leaders and parent educators will complete data collection digitally, including for visit records; this is consistent with usual PAT practice. PAT collects existing, routine reporting on visits with families. As the visit records are extra reporting for research purposes beyond the existing reporting, parent educators will receive $10 incentives for each visit record completed. For parent educators at sites randomized to HEALTH, surveys to assess RE-AIM metrics will be administered before and after the initial training component of the HEALTH training curriculum, then again at the end of the intervention period (after the 18 mothers at the site have completed the 2-year follow-up). Parent educators at sites randomized to usual care will complete a first set of surveys at enrollment, then again before and after receiving the delayed HEALTH training curriculum (after all mothers at the site have completed the 2-year follow-up). In prior work with PAT staff, 83% of parent educators and 80% of site leaders completed online surveys [96]. The survey link will be emailed to the PAT staff member. Upon completion of the survey, respondents will receive a $15 gift card. All data collection will occur through REDCap (Research Electronic Data Capture), which is a mature, secure web application for building and managing online surveys and databases [97]. Random checks of data collection activities will assure reliability.

### Qualitative data collection procedures

Mothers, parent educators, and PAT site leaders will be purposively selected to be representative of those with varying (high, middle, low) scores on the quantitative measures for acceptability. Interviews will be digitally recorded. If the participant is not reached on the first attempt, they will be called three times on varying days/times of day. Before the interview, the interviewer will read consent information and obtain verbal permission to participate and record the interview; the interviewer will mail or email consent information sheets, based on participant preference. We anticipate interviews will last 45–60 min. Implementation outcomes in the RE-AIM domains Adoption and Implementation will be collected using interview guides informed by Blaine et al. for acceptability, appropriateness, and feasibility [79] and by Wiltsey Stirman et al. for adaptation [51, 85]. For mothers, interviews will be conducted after they have completed HEALTH. Parent educators will complete interviews after conducting HEALTH with at least three families. Though a formal power analysis is not appropriate for qualitative studies, research indicates six interviews should be adequate to reach data saturation [98]; we anticipate including 15 mothers, 15 HEALTH parent educators, and 10 site leaders, but will conduct interviews until we reach thematic saturation. Upon completion of the interview, participants will receive a $30 gift card.

### Sample size and statistical power

The sample size for this cluster randomized study (28 sites (14 to each arm), 18 women per site) provides a statistical power of 0.9 with 12 mothers completing 24 months of follow-up at each of 24 PAT sites with two-sided tests at the 0.05 significance level to detect the magnitude of the between-group difference in the baseline to 24-month change in body weight of 4.77, with an intraclass correlation coefficient of 0.05, and a standard deviation of 10. The HEALTH effectiveness trial found the standard deviation of the change in weight from baseline to 24 months was 8.3 kg; however, to be conservative, we assume a standard deviation of 10. The computations also assume 12 mothers will complete 24 months of follow-up at each of 24 PAT sites, accounting for a potential dropout rate of 32% for mothers and loss of two sites per arm; this includes exclusion of mothers who become pregnant during the course of the study. The sample size also allows for attrition of two study sites. The HEALTH study saw a 24-month decrease in weight of 1.5 (± 8.3) kg in the intervention group and a 24-month increase of 3.2 (± 7.6) kg in the control. Our analysis will also add power by including data on the dropouts who do not provide 24-month data but who do provide data at 12 months.

### Statistical analyses

The primary analysis will be a mixed model repeated measures analysis of variance (ANOVA) with the mother nested within the PAT site and with the outcome variable, body weight, quantified as a continuous variable. In addition, we may employ nested analysis of covariance (ANCOVA) models that adjust for covariates (e.g., income), if these are not balanced by randomization. Secondary endpoints such as daily intake of sugary beverages and the Household Food Practices and Family Social Support will be evaluated as count variables which, depending on the distributional properties of the count, will be evaluated using Poisson regression or negative binomial regression if the Poisson model does not fit. The outcome variables for the assessment of implementation outcomes, fidelity (primary) and other implementation outcomes (e.g., acceptability) (secondary), will be treated as continuous variables and will be analyzed using the same nested mixed models discussed for the weight outcomes.

To address the exploratory external validity questions, we will use data provided from PAT National Center to investigate demographic and socio-economic characteristics between mothers in HEALTH and typical PAT mothers with *t* tests and chi-square tests to compare between groups and determine representativeness [99]. We will use multilevel modeling (or nested linear modeling) to determine whether parent educator characteristics, as measured by CFIR constructs (e.g., self-efficacy) are predictors of fidelity (a continuous variable), while adjusting for clustering by site. Similar methods will be used to determine predictors of adoption at the site level using inner and outer setting characteristics from CFIR.

Qualitative analysis will be conducted of the digital recordings, which will be transcribed. Two researchers will analyze the transcripts. For the adaptation analysis, coding will be guided by the work of Wiltsey Stirman et al. [51, 85] using a combination of deductive and emergent coding [100]. For all other analyses, after reviewing the research questions [101, 102], team members will read five transcripts using the first draft of a codebook. Each coder will systematically review the data and organize each statement into categories that summarize the concept or meaning [103]. Once these transcripts are coded, they will be discussed to ensure accuracy of the codebook and inter-coder consistency. The codebook will be edited as needed prior to coding the remaining transcripts. All transcripts will be analyzed by two coders in NVivo11; disagreements will be resolved by discussion with study team members [104]. Themes from coded transcripts will be summarized and highlighted with exemplary quotes.

## Discussion

Embedding intervention content within existing delivery channels can help expand the reach of evidence-based interventions. Reaching hard to reach populations may require deviation from prescribed intensity and/or dose from original interventions such as the DPP. Interventions, which have been adapted, can still be effective even if the effect is reduced and can still achieve population impact by reaching a broader set of the population. The current study will build on this to test not only the effectiveness of HEALTH in real-world PAT implementation nationwide, but also elements critical to D&I, implementation outcomes, and the context for implementation. Recognizing the importance of evaluating an intervention not only for its impact on weight, but also for its ability to be implemented in real-world settings and to reach mothers often left out of other lifestyle modification programs is a critical application of D&I science.

There are potential limitations and methodological decisions to consider with the current study. The overarching methodological decision for this study is the Hybrid Type 2 nature of the design, which aims to balance questions of efficacy/effectiveness (internal validity) and D&I (external validity). While this is an important strength as the findings related to implementation outcomes, context, and external validity are critical to the future impact of the HEALTH intervention, there is an inherent trade-off with internal validity. We summarize the relative pragmatic and explanatory [105] aspects of the study using the PRagmatic Explanatory Continuum Indicator Summary (PRECS-2) scheme [106, 107], which is a tool to help with study design considerations. Additional file 1: Table S2 provides detailed definitions of the domains along with the scores; briefly, on the nine PRECS-2 domains, the team ranked the study as eligibility criteria [5], recruitment [5], setting [5], organization [5], flexibility delivery [5], flexibility adherence [5], follow-up [5], primary outcome [3], and primary analysis [4]. Also, due to the pragmatic nature of the study, inclusion criteria have been set as broadly as possible, which may lead to a study population that is quite heterogeneous; while this may reduce our ability to detect an effect or reduce the effect observed, it will enhance the ability of the study to demonstrate the true impact of HEALTH when implemented in real-world practice and enhance the generalizability of the findings to allow for greater use.

In conclusion, this study will provide valuable data related to effectiveness and D&I of an evidence-based intervention to prevent weight gain among young mothers. This is a particularly important population, as women in this group are often difficult to reach with traditional lifestyle management interventions, yet are at very high risk for excessive weight gain. The partnership with PAT and delivery of the HEALTH training curriculum through PAT National Center’s existing training infrastructure and embedded content enhance the potential for D&I and widespread impact on weight and chronic disease outcomes.

## Additional file

Additional file 1: **Table S1.** Overview of the Healthy Eating and Active Living Taught at Home (HEALTH) intervention. **Table S2.** Definition of PRECIS-2 Domains and scores for current trial. (DOCX 20 kb)

## Data Availability

The datasets used and/or analyzed during the current study are available from the corresponding author on reasonable request and appropriate human subject approval.

## Abbreviations

ANCOVA: Analysis of covariance
ANOVA: Analysis of variance
CFIR: Conceptual Framework for Implementation Research
D&I: Dissemination and implementation
DPP: Diabetes Prevention Program
HEALTH: Healthy Eating and Active Living Taught at Home
NHANES: National Health and Nutrition Examination Survey
PAT: Parents as Teachers
PRECS-2: PRagmatic Explanatory Continuum Indicator Summary
RE-AIM: Reach, Efficacy, Adoption, Implementation, Maintenance

## References

[1] Dutton GR, Kim Y, Jacobs DR, Li X, Loria CM, Reis JP (2016). 25-year weight gain in a racially balanced sample of U.S. adults: the CARDIA study. Obesity..

[2] Lewis CE, Jacobs DR, McCreath H, Kiefe CI, Schreiner PJ, Smith DE (2000). Weight gain continues in the 1990s: 10-year trends in weight and overweight from the CARDIA study. Coronary Artery Risk Development in Young Adults. Am J Epidemiol.

[3] Dietz WH (2017). Obesity and excessive weight gain in young adults: new targets for prevention. JAMA..

[4] Harrison CL, Skouteris H, Boyle J, Teede HJ (2017). Preventing obesity across the preconception, pregnancy and postpartum cycle: implementing research into practice. Midwifery..

[5] Walker LO, Sterling BS, Kim M, Arheart KL, Timmerman GM (2006). Trajectory of weight changes in the first 6 weeks postpartum. J Obst Gynecol Neonatal Nurs.

[6] Williamson D, Madans J, Pamuk E, Flegal K, Kendrick J, Serdula M (1994). A prospective study of childbearing and 10-year weight gain in US white women 25 to 45 years of age. Int J Obes.

[7] National Center for Health Statistics (NCHS). National Health and nutrition examination survey Atlanta, GA2016. Available from: https://www.cdc.gov/nchs/data/factsheets/factsheet_nhanes.pdf. [Accessed 17 June 2019].

[8] Flegal KM, Kruszon-Moran D, Carroll MD, Fryar CD, Ogden CL (2016). Trends in obesity among adults in the United States, 2005 to 2014. JAMA..

[9] Zheng Y, Manson JE, Yuan C, Liang MH, Grodstein F, Stampfer MJ (2017). Associations of weight gain from early to middle adulthood with major health outcomes later in life. JAMA..

[10] Williamson DF, Kahn HS, Remington PL, Anda RF (1990). The 10-year incidence of overweight and major weight gain in US adults. Arch Intern Med.

[11] Haire-Joshu D, Schwarz CD, Steger-May K, Lapka C, Schechtman K, Brownson RC (2018). A randomized trial of weight change in a national home visiting program. Am J Prev Med.

[12] Knowler WC, Barrett-Connor E, Fowler SE, Hamman RF, Lachin JM, Walker EA (2002). Reduction in the incidence of type 2 diabetes with lifestyle intervention or metformin. N Engl J Med.

[13] Diabetes Prevention Program Research Group (2015). Long-term effects of lifestyle intervention or metformin on diabetes development and microvascular complications over 15-year follow-up: the Diabetes Prevention Program Outcomes Study. Lancet Diabetes Endocrinol.

[14] Wadden TA, Anderson DA, Foster GD (1999). Two-year changes in lipids and lipoproteins associated with the maintenance of a 5% to 10% reduction in initial weight: some findings and some questions. Obes Res.

[15] Stefanick ML, Mackey S, Sheehan M, Ellsworth N, Haskell WL, Wood PD (1998). Effects of diet and exercise in men and postmenopausal women with low levels of HDL cholesterol and high levels of LDL cholesterol. N Engl J Med.

[16] Dattilo AM, Kris-Etherton PM (1992). Effects of weight reduction on blood lipids and lipoproteins: a meta-analysis. Am J Clin Nutr.

[17] The Trials of Hypertension Prevention Collaborative Research Group (1997). Effects of weight loss and sodium reduction intervention on blood pressure and hypertension incidence in overweight people with high-normal blood pressure. The Trials of Hypertension Prevention, phase II. Arch Intern Med.

[18] Aziz Z, Absetz P, Oldroyd J, Pronk NP, Oldenburg B (2015). A systematic review of real-world diabetes prevention programs: learnings from the last 15 years. Implement Sci.

[19] Johnson M, Jones R, Freeman C, Woods HB, Gillett M, Goyder E (2013). Can diabetes prevention programmes be translated effectively into real-world settings and still deliver improved outcomes? A synthesis of evidence. Diabet Med.

[20] Ackermann RT, Marrero DG (2007). Adapting the diabetes prevention program lifestyle intervention for delivery in the community: the YMCA model. Diabetes Educ.

[21] Neamah HH, Sebert Kuhlmann AK, Tabak RG (2016). Effectiveness of program modification strategies of the diabetes prevention program: a systematic review. Diabetes Educ..

[22] Tabak RG, Sinclair KA, Baumann AA, Racette SB, Sebert Kuhlmann A, Johnson-Jennings MD (2015). A review of diabetes prevention program translations: use of cultural adaptation and implementation research. Transl Behav Med.

[23] Whittemore R (2011). A systematic review of the translational research on the Diabetes Prevention Program. Transl Behav Med.

[24] Ritchie ND, Sauder KA, Fabbri S. Reach and effectiveness of the National Diabetes Prevention Program for young women. Am J Prev Med. 2017; in press. 10.1016/j.amepre.2017.06.013 28928038

[25] Ali MK, Echouffo-Tcheugui J, Williamson DF (2012). How effective were lifestyle interventions in real-world settings that were modeled on the diabetes prevention program?. Health Aff.

[26] Kirchner JE, Waltz TJ, Powell BJ, Smith, J. L, Proctor EK.Implementation strategies. In: Brownson R, Colditz G, Proctor E, editors. Dissemination and implementation research in health: translating science to practice. 2. New York: Oxford University Press; 2018. p. 245–266.

[27] Bauer MS, Damschroder L, Hagedorn H, Smith J, Kilbourne AM (2015). An introduction to implementation science for the non-specialist. BMC Psychol.

[28] Proctor EK, Powell BJ, McMillen JC (2013). Implementation strategies: recommendations for specifying and reporting. Implement Sci.

[29] Curran GM, Bauer M, Mittman B, Pyne JM, Stetler C (2012). Effectiveness-implementation hybrid designs: combining elements of clinical effectiveness and implementation research to enhance public health impact. Med Care.

[30] Murray DM, Varnell SP, Blitstein JL (2004). Design and analysis of group-randomized trials: a review of recent methodological developments. Am J Public Health.

[31] Murray DM (1998). Design and analysis of group-randomized trials.

[32] Varnell SP, Murray DM, Janega JB, Blitstein JL (2004). Design and analysis of group-randomized trials: a review of recent practices. Am J Public Health.

[33] Glanz K, Rimer BK, Lewis FM (2002). Health behavior and health education: theory, research, and practice.

[34] Sallis J, Owen N, Glanz K, Lewis FM, Rimer BK (1997). Ecological models of health behavior. Health behavior and health education theory, research, and practice.

[35] Baranowski T, Perry CL, Parcel G, Glanz K, Rimer B, Lewis F (1997). How individuals, environments, and health behavior interact: social cognitive theory. Health behavior and health education: theory, research, practice.

[36] Nilsen P (2015). Making sense of implementation theories, models and frameworks. Implement Sci.

[37] CFIR Research Team. Consolidated Framework for Implementation Research (CFIR) [Available from: http://cfirguide.org/. Accessed 17 June 2019.

[38] Damschroder LJ, Aron DC, Keith RE, Kirsh SR, Alexander JA, Lowery JC (2009). Fostering implementation of health services research findings into practice: a consolidated framework for advancing implementation science. Implement Sci.

[39] Rogers EM (2003). Diffusion of innovations.

[40] Greenhalgh T, Robert G, Macfarlane F, Bate P, Kyriakidou O (2004). Diffusion of innovations in service organizations: systematic review and recommendations. Milbank Q.

[41] Dzewaltowski DA, Estabrooks PA, Klesges LM, Bull S, Glasgow RE (2004). Behavior change intervention research in community settings: how generalizable are the results?. Health Promot Int.

[42] Glasgow RE, Nelson CC, Strycker LA, King DK (2006). Using RE-AIM metrics to evaluate diabetes self-management support interventions. Am J Prev Med.

[43] Gary TL, Hill-Briggs F, Batts-Turner M, Brancati FL (2005). Translational research principles of an effectiveness trial for diabetes care in an urban African American population. Diabetes Educ.

[44] Glasgow RE (2006). RE-AIMing research for application: ways to improve evidence for family medicine. J Am Board Fam Med.

[45] Swinburn B, Gill T, Kumanyika S (2005). Obesity prevention: a proposed framework for translating evidence into action. Obes Rev.

[46] Bopp M, Wilcox S, Laken M, Hooker SP, Saunders R, Parra-Medina D (2007). Using the RE-AIM framework to evaluate a physical activity intervention in churches. Prev Chronic Dis.

[47] Glasgow RE, Klesges LM, Dzewaltowski DA, Estabrooks PA, Vogt TM (2006). Evaluating the impact of health promotion programs: using the RE-AIM framework to form summary measures for decision making involving complex issues. Health Educ Res.

[48] Jilcott S, Ammerman A, Sommers J, Glasgow RE (2007). Applying the RE-AIM framework to assess the public health impact of policy change. Ann Behav Med.

[49] Dzewaltowski DA, Glasgow RE, Klesges LM, Estabrooks PA, Brock E (2004). RE-AIM: evidence-based standards and a Web resource to improve translation of research into practice. Ann Behav Med.

[50] Scott SD, Plotnikoff RC, Karunamuni N, Bize R, Rodgers W (2008). Factors influencing the adoption of an innovation: an examination of the uptake of the Canadian Heart Health Kit (HHK). Implement Sci.

[51] Stirman SW, Miller CJ, Toder K, Calloway A (2013). Development of a framework and coding system for modifications and adaptations of evidence-based interventions. Implement Sci.

[52] Toobert DJ, Strycker LA, Glasgow RE, Bagdade JD (2002). If you build it, will they come?. Reach and adoption associated with a comprehensive lifestyle management program for women with type 2 diabetes. Patient Educ Couns.

[53] Moore JE, Mascarenhas A, Bain J, Straus SE (2017). Developing a comprehensive definition of sustainability. Implement Sci.

[54] Ford I, Norrie J (2016). Pragmatic trials. N Engl J Med.

[55] Pocock SJ, Simon R (1975). Sequential treatment assignment with balancing for prognostic factors in the controlled clinical trial. Biometrics..

[56] Parents as Teachers National Center. PARENTS AS TEACHERS. 2019. Retrieved from https://parentsasteachers.org/. Accessed 18 June 2019.

[57] Parents as Teachers National Center. TRAINING & CURRICULA - PARENTS AS TEACHERS. 2019. Retrieved from https://parentsasteachers.org/training-curricula. Accessed 18 June 2019.

[58] Diabetes Prevention Program Research G, Knowler WC, Fowler SE, Hamman RF, Christophi CA, Hoffman HJ, et al. 10-year follow-up of diabetes incidence and weight loss in the diabetes prevention program outcomes study. Lancet. 2009;374(9702):1677–1686. 10.1016/S0140-6736(09)61457-4 PMC3135022 19878986

[59] Powell BJ, Waltz TJ, Chinman MJ, Damschroder LJ, Smith JL, Matthieu MM (2015). A refined compilation of implementation strategies: results from the Expert Recommendations for Implementing Change (ERIC) project. Implement Sci.

[60] Haire-Joshu D, Elliott MB, Caito NM, Hessler K, Nanney MS, Hale N (2008). High 5 for kids: the impact of a home visiting program on fruit and vegetable intake of parents and their preschool children. Prev Med.

[61] Haire-Joshu D, Nanney MS, Elliott M, Davey C, Caito N, Loman D (2010). The use of mentoring programs to improve energy balance behaviors in high-risk children. Obesity..

[62] Haire-Joshu DL, Schwarz CD, Peskoe SB, Budd EL, Brownson RC, Joshu CE (2015). A group randomized controlled trial integrating obesity prevention and control for postpartum adolescents in a home visiting program. Int J Behav Nutr Phys Act.

[63] Craig CL, Marshall AL, Sjostrom M, Bauman AE, Booth ML, Ainsworth BE (2003). International physical activity questionnaire: 12-country reliability and validity. Med Sci Sports Exerc.

[64] Kegler MC, Alcantara I, Haardorfer R, Gazmararian JA, Ballard D, Sabbs D (2014). The influence of home food environments on eating behaviors of overweight and obese women. J Nutr Educ Behav.

[65] CDC NHANES. Center for Disease Control and Prevention. National health and nutrition examination survey: anthropometry procedures manual 2016 [Available from: https://www.cdc.gov/nchs/data/nhanes/nhanes_15_16/2016_Anthropometry_Procedures_Manual.pdf. Accessed 17 June 2019.

[66] Lee K (2008). Waist circumference percentile criteria for the pediatric metabolic syndrome in Korean adolescents. Asia Pac J Clin Nutr.

[67] Fernandez JR, Redden DT, Pietrobelli A, Allison DB (2004). Waist circumference percentiles in nationally representative samples of African-American, European-American, and Mexican-American children and adolescents. J Pediatr.

[68] Huxley R, Mendis S, Zheleznyakov E, Reddy S, Chan J (2010). Body mass index, waist circumference and waist:hip ratio as predictors of cardiovascular risk--a review of the literature. Eur J Clin Nutr.

[69] Esteghamati A, Ashraf H, Rashidi A, Meysamie A (2008). Waist circumference cut-off points for the diagnosis of metabolic syndrome in Iranian adults. Diabetes Res Clin Pract.

[70] Mailey EL, Rosenkranz SK, Casey K, Swank A (2016). Comparing the effects of two different break strategies on occupational sedentary behavior in a real world setting: a randomized trial. Prev Med Rep.

[71] Zoellner J, Chen Y, Davy B, You W, Hedrick V, Corsi T (2014). Talking health, a pragmatic randomized-controlled health literacy trial targeting sugar-sweetened beverage consumption among adults: rationale, design & methods. Contemp Clin Trials.

[72] Centers for Disease Control and Prevention National Health and Nutrition Examination Survey. Dietary screener questionnaire 2009–2010 [Available from: https://epi.grants.cancer.gov/nhanes/dietscreen/. Accessed 17 June 2019.

[73] Aarons GA (2004). Mental health provider attitudes toward adoption of evidence-based practice: the Evidence-Based Practice Attitude Scale (EBPAS). Ment Health Serv Res.

[74] Massatti RR, Sweeney HA, Panzano PC, Roth D (2008). The de-adoption of innovative mental health practices (IMHP): why organizations choose not to sustain an IMHP. Admin Pol Ment Health.

[75] Panzano PC, Sweeney HA, Seffrin B, Massatti R, Knudsen KJ (2012). The assimilation of evidence-based healthcare innovations: a management-based perspective. J Behav health Serv Res.

[76] Pankratz M, Hallfors D, Cho H (2002). Measuring perceptions of innovation adoption: the diffusion of a federal drug prevention policy. Health Educ Res.

[77] Calhoun A, Mainor A, Moreland-Russell S, Maier RC, Brossart L, Luke DA (2014). Using the program sustainability assessment tool to assess and plan for sustainability. Prev Chronic Dis.

[78] Luke DA, Calhoun A, Robichaux CB, Elliot MB, Moreland-Russell S (2014). The program sustainability assessment tool: a new instrument for public health programs. Prev Chronic Dis.

[79] Blaine RE, Franckle RL, Ganter C, Falbe J, Giles C, Criss S (2017). Using school staff members to implement a childhood obesity prevention intervention in low-income school districts: the Massachusetts Childhood Obesity Research Demonstration (MA-CORD project), 2012-2014. Prev Chronic Dis.

[80] Steckler A, Goodman RM, McLeroy KR, Davis S, Koch G (1992). Measuring the diffusion of innovative health promotion programs. Am J Health Promot.

[81] Glasgow RE, Green LW, Klesges LM, Abrams DB, Fisher EB, Goldstein MG (2006). External validity: we need to do more. Ann Behav Med.

[82] Klesges LM, Dzewaltowski DA, Glasgow RE (2008). Review of external validity reporting in childhood obesity prevention research. Am J Prev Med.

[83] Glasgow RE, Klesges LM, Dzewaltowski DA, Bull SS, Estabrooks P (2004). The future of health behavior change research: what is needed to improve translation of research into health promotion practice?. Ann Behav Med.

[84] Chambers DA, Glasgow RE, Stange KC (2013). The dynamic sustainability framework: addressing the paradox of sustainment amid ongoing change. Implement Sci.

[85] Wiltsey Stirman S, Calloway A, Toder K, Miller CJ, Devito AK, Meisel SN (2013). Community mental health provider modifications to cognitive therapy: implications for sustainability. Psychiatr Serv.

[86] Baumann AA, Cabassa LJ, Adaptation in Dissemination WSS, Brownson R, Colditz G, Proctor E (2018). Implementation Science. Dissemination and implementation research in health: translating science to practice.

[87] Aarons GA, Palinkas LA (2007). Implementation of evidence-based practice in child welfare: service provider perspectives. Admin Pol Ment Health.

[88] Palinkas LA, Schoenwald SK, Hoagwood K, Landsverk J, Chorpita BF, Weisz JR (2008). An ethnographic study of implementation of evidence-based treatments in child mental health: first steps. Psychiatr Serv.

[89] Stirman SW, Kimberly J, Cook N, Calloway A, Castro F, Charns M (2012). The sustainability of new programs and innovations: a review of the empirical literature and recommendations for future research. Implement Sci.

[90] Chambers DA, Norton WE (2016). The adaptome: advancing the science of intervention adaptation. Am J Prev Med.

[91] Latimer AE, Martin Ginis KA (2005). The importance of subjective norms for people who care what others think of them. Psychol Health.

[92] Helfrich CD, Li YF, Sharp ND, Sales AE (2009). Organizational readiness to change assessment (ORCA): development of an instrument based on the Promoting Action on Research in Health Services (PARIHS) framework. Implement Sci.

[93] Fernandez ME, Walker TJ, Weiner BJ, Calo WA, Liang S, Risendal B (2018). Developing measures to assess constructs from the inner setting domain of the consolidated framework for implementation research. Implement Sci.

[94] Shea CM, Jacobs SR, Esserman DA, Bruce K, Weiner BJ (2014). Organizational readiness for implementing change: a psychometric assessment of a new measure. Implement Sci.

[95] Yun D, Silk KJ (2011). Social norms, self-identity, and attention to social comparison information in the context of exercise and healthy diet behavior. Health Commun.

[96] Tabak R, Schwarz C, Carter E, Haire-Joshu D (2018). Context for implementing a gestational weight gain program nationally. Health Behav Policy Rev.

[97] Harris PA, Taylor R, Thielke R, Payne J, Gonzalez N, Conde JG (2009). Research electronic data capture (REDCap)--a metadata-driven methodology and workflow process for providing translational research informatics support. J Biomed Inform.

[98] Curry LA, Nembhard IM, Bradley EH (2009). Qualitative and mixed methods provide unique contributions to outcomes research. Circulation..

[99] Parents As Teachers (2015). Annual Report 2014-2015.

[100] Stuckey HL (2015). The second step in data analysis: coding qualitative research data. J Soc Health Diabetes.

[101] Strauss A (1987). Qualitative analysis for social scientists.

[102] Strauss A, Corbin J (1998). Basics of qualitative research: second edition: techniques and procedures for developing grounded theory.

[103] Patton MQ (2002). Qualitative evaluation and research methods.

[104] QSR International. NVivo10 Victoria, Australia [Available from: http://www.qsrinternational.com/product. Accessed 17 June 2019.

[105] Thorpe KE, Zwarenstein M, Oxman AD, Treweek S, Furberg CD, Altman DG (2009). A pragmatic-explanatory continuum indicator summary (PRECIS): a tool to help trial designers. J Clin Epidemiol.

[106] Loudon K, Treweek S, Sullivan F, Donnan P, Thorpe KE, Zwarenstein M (2015). The PRECIS-2 tool: designing trials that are fit for purpose. BMJ.

[107] Loudon K, Zwarenstein M, Sullivan FM, Donnan PT, Gagyor I, Hobbelen H (2017). The PRECIS-2 tool has good interrater reliability and modest discriminant validity. J Clin Epidemiol.

