# Supplementary material for: Disseminating and implementing a lifestyle-based healthy weight program for mothers in a national organization: a study protocol for a cluster randomized trial
Source: Implement Sci. 2019 Jun 25;14:68. doi: 10.1186/s13012-019-0916-0 (PMC6593605; doi:10.1186/s13012-019-0916-0)
Supplement: Supplementary file 1 — Table S1. Overview of the Healthy Eating and Active Living Taught at Home (HEALTH) intervention. Table S2. Definition of PRECIS-2 Domains and scores for current trial. (DOCX 20 kb) [file 13012_2019_916_MOESM1_ESM.docx]

Table S1. Overview of the Healthy Eating and Active Living Taught at Home (HEALTH) intervention

|  | HEALTH Topic | Sample Intent | Sample Prompt/question |
| --- | --- | --- | --- |
| Core Visits (1^st^ 4-6 months - ideally every visit with family) | 1. Welcome to HEALTH and goal setting ) | Welcome to HEALTH. | Why did you want to participate in this study? |
|  | 1. Healthy beverages | Build skills in reading labels to discuss sugar content and calories in various beverages. | When you are thirsty, what do you drink? |
|  | 1. Being active: a way of life | Identify barriers to being active. | Is there anything that makes it hard for you to be active? |
|  | 1. Self-monitoring | Increase parent’s awareness of their actions and how to track their behaviors. | Have you ever tracked what you have eaten or drank? If so, why? |
|  | 1. Portion Size | Build skills in choosing healthy portions. | What size plates do you use during meals? |
|  | 1. Cope with triggers | Provide an opportunity to learn about food cues and ways to change them. | What makes you want to eat? |
|  | 1. Eat well away from home | Practice how to apply these principles at the type of restaurant the participant frequents. | What places do you frequent when you eat away from home? |
|  | 1. Meal planning | Provide an opportunity for parents to brainstorm meals that their family eats. | How does your family decide what to eat at a meal? |
| Maintenance Visits (at least monthly) | 1. Cook More | Discuss practical tips to encourage more cooking at home. | What are the kid(s) doing when you are cooking? |
|  | 1. Healthy snacking | Build skills in measuring out appropriate portions of food. | Can we look at a typical food that you consider a snack? |
|  | 1. Family Meals | Plan and coordinate family meals without distractions (TV). | Where do you eat meals? |
|  | 1. Ways to eat less calories | Identify ways to reduce calories. | What are some foods that you think are problematic? |
|  | 1. Find time for fitness | Provide an opportunity for parents to identify positive cues to be active at home. | What type of activities do you do with your kids? |
|  | 1. Problem solving | Learn the steps to problem solving. | What problems have you encountered while striving to be healthy? |
|  | 1. Get support | Explore examples of problematic social cues and helpful social cues. | When you are working towards your goal how do other people effect your progress? |
|  | 1. You can manage stress | Provide an opportunity for parents to discuss and identify stressors in their life. | When you are stressed, what do you do? |
|  | 1. Healthy breakfast | Identify strategies to eat a healthy breakfast. | What happens if you don’t eat breakfast? |
|  | 1. Healthy routines | Brainstorm and discuss routines in everyday life. | Do you have any regular eating routines? |
|  | 1. Food labels | Build skills in reading nutrition-fact labels. | Do you make purchases based on the nutrition information from the food labels? |
|  | 1. Jump start your activity plan | Provide an opportunity for parents to acknowledge and identify barriers to meeting their goals. | What has made it hard to meet your activity goals? |
|  | 1. Take charge of your thoughts/Ways to avoid burnout | Assist parents in learning how to talk back to negative thoughts with positive thoughts. | What strategies have worked for you when you feel discouraged? |
|  | 1. Shopping | Build skills in creating lists to grocery shop for planned meals. | What pulls your attention when you are at the grocery store? |
|  | 1. Stay motivated | Discuss the importance of motivation and ways to stay motivated. | What helped you stay on track with your goals? |

| Table S2. Definition of PRECIS-2 Domains and scores for current trial | | |
| --- | --- | --- |
| PRECIS-2 Domain | Score | Explanation of Domain |
| Eligibility criteria | 4 | To what extent are the participants in the trial similar to those who would receive this intervention if it was part of usual care? |
| Recruitment | 5 | How much extra effort is made to recruit participants over and above what would be used in the usual care setting to engage with patients? |
| Setting | 5 | How different are the settings of the trial from the usual care setting? |
| Organization | 5 | How different are the resources, provider expertise, and the organization of care delivery in the intervention arm of the trial from those available in usual care? |
| Flexibility (delivery) | 5 | How different is the flexibility in how the intervention is delivered and the flexibility anticipated in usual care? |
| Flexibility (adherence) | 5 | How different is the flexibility in how participants are monitored and encouraged to adhere to the intervention from the flexibility anticipated in usual care? |
| Follow-up | 5 | How different is the intensity of measurement and follow-up of participants in the trial from the typical follow-up in usual care? |
| Primary outcome | 3 | To what extent is the trial’s primary outcome directly relevant to participants? |
| Primary analysis | 4 | To what extent are all data included in the analysis of the primary outcome? |

Definitions are from Loudon Kirsty, Treweek Shaun, Sullivan Frank, Donnan Peter, Thorpe Kevin E, Zwarenstein Merrick et al. The PRECIS-2 tool: designing trials that are fit for purpose *BMJ* 2015; 350 :h2147. Additional details about scoring (i.e., what increases or decreases scores) are available in the article and the PRECIS-2 website: <https://www.precis-2.org/>

PRECIS = PRagmatic Explanatory Continuum Indicator Summary
